# Taken to School: An Interview with the Honorable Judge John E. Jones, III

**DOI:** 10.1371/journal.pgen.1000297

**Published:** 2008-12-05

**Authors:** Jane Gitschier

**Affiliations:** Departments of Medicine and Pediatrics, Institute for Human Genetics, University of California San Francisco, San Francisco, California, United States of America

I once had a post-doctoral fellow who, upon discovering we had grown up a quarter mile from each other in a small town in Pennsylvania, commented on our shared experience with, “Well, you know what they say about Pennsylvania? There's Philadelphia, there's Pittsburgh, and there's the state of Alabama in between.” That blunt assessment (attributable to James Carville) certainly resonated when I first read about the Kitzmiller et al. v. Dover Area School District case in late 2004.

Dover, indeed, is a small town in south central Pennsylvania. At that time, the Dover school board instructed 9th grade biology teachers to read a statement that evolution is only a theory for the origin of species and to proffer an alternate explanation called “intelligent design” (ID). Tammy Kitzmiller, the mother of two students in the Dover public schools, together with a number of other plaintiffs and assisted by the American Civil Liberties Union (ACLU), sued the Dover school district for an injunction against the statement and use of materials in science class as a breach of the First Amendment of the US Constitution.

**Image 1:**
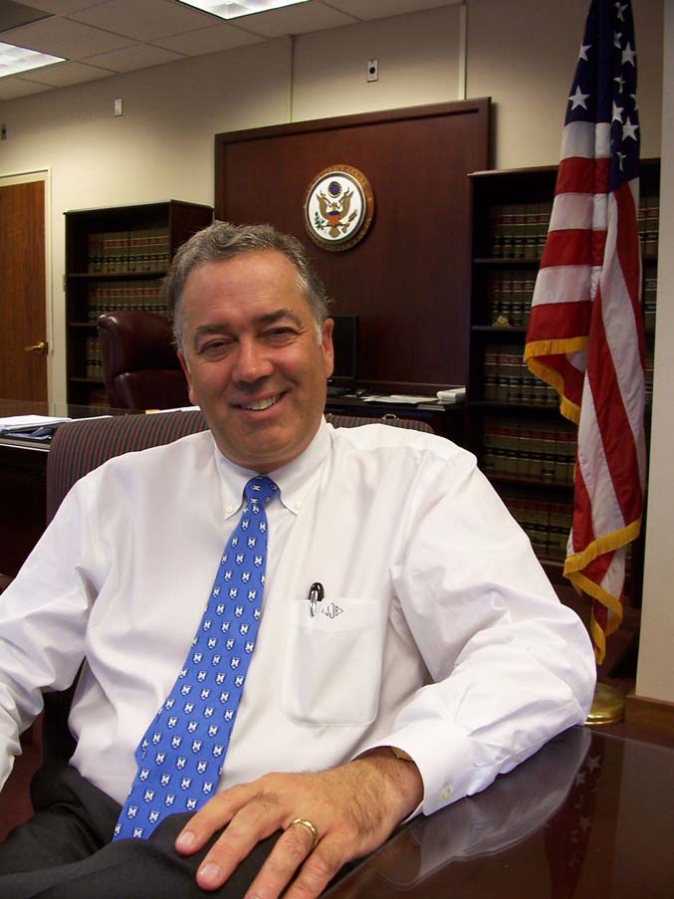
Judge John E. Jones, III.

At the bench during this remarkable trial sat the Federal Judge for the Middle District of Pennsylvania, the Honorable Judge John E. Jones, III (see Image 1). Jones, a Republican and a Bush appointee, was assumed by many and feared by others to be inclined to rule for the defendants. However, in a stunning Memorandum Opinion (see http://www.pamd.uscourts.gov/kitzmiller/kitzmiller_342.pdf), Jones excoriated intelligent design, waxed eloquent about the meaning and practice of science, and, for the skeptics, restored faith in the fairness of the judicial system.

My call to the Judge's chambers in request for an interview was answered in vivo by his assistant, who suggested simply e-mailing the Judge directly. I did, and back came an immediate reply of “Happy to do it.” On the appointed July day, in near 100-degree heat, I drove from my father's home in Pottstown along country roads through the corn-laden, cow-dotted agricultural landscape that I love. But as I got closer to my destination, the state capital of Harrisburg, billboard outcroppings disrupted the fields' quiet beauty with warnings such as, “It's your choice – heaven or hell.” It appeared that I had arrived at the crux of the matter.

**Gitschier:** I am very excited to meet you. There are roughly three areas I want to talk to you about.

**Jones:** Do my best.

**Gitschier:** One has to do with your background—your thinking about evolution, intelligent design, creationism—going into the trial, your experience during the trial, and then afterwards—how this might have changed you.

The second is to help me through the legal stuff. I'm not a lawyer and I'm going to be writing this for an audience of geneticists.

The third is a shorter question—the ramifications of this decision on public education in the US.

So, let's first cover a little background.

**Jones:** I'm from Pottsville, PA, which is in the anthracite coal region of northeast central Pennsylvania. And I was raised in Orwigsburg, a little town of about 2,000 not far from there. It's in an old industrial coal county. I went to Dickinson College and Dickinson School of Law, and returned there to practice. My family roots are very deep there. It occurred to me that I'd probably be able to start a successful law practice back there and I was, happily, right about that.

**Gitschier:** What kind of law did you practice?

**Jones:** I was a general practitioner, which increasingly is a dinosaur. I used to say that I was a half-an-inch deep and a half-mile wide. I needed to know a little about a lot of different things. I was the quintessential country lawyer.

**Gitschier:** So—wills, small disputes?

**Jones:** Everything. I did a lot of litigation. I liked to go to court. I became a lawyer because of the allure of the courtroom, not necessarily to be chained to an office desk.

**Gitschier:** I'd like to deal with some of the legal stuff I don't understand. Kitzmiller was a suit. What does that mean? I usually think of suing for money or restitution.

**Jones:** That's a very good question. There is a statute, known as section 1983, in the Federal Law, and in layman's terms, it's an enabling statute and it allows you to bring suit in federal court if you believe that a constitutional right has been violated. And notably, in the context of the Dover case, it allows you to recover your fees and costs if you prevail.

When this suit was brought in December of 2004, although the statute also allows you to seek money damages, that was not the request. The request was for an injunction. An injunction is a legal ruling that stops something, typically, from happening. The plaintiffs asked for an injunction to stop the policy from being implemented in the first instance. It was to be implemented in January of 2005 after it had been enacted in 2004.

That's why it was a bench trial, and not a jury trial, to anticipate a question you may have, because when you ask for an injunction, only a judge can grant an injunction. Had they [the plaintiffs] asked for money damages, it would have been brought to a jury. They were never interested, it appeared to me, in money damages. They were interested in stopping the policy from being implemented. That was their real goal throughout the litigation.

**Gitschier:** You're right, I was going to ask you about why this was a bench trial.

**Jones:** Everybody does.

**Gitschier:** Because the Scopes trial [in 1925] was a jury trial.

**Jones:** Well, that was a criminal prosecution. John Scopes was prosecuted under a Tennessee statute, which had been little used, that prohibited the teaching of evolution.

**Gitschier:** Little used because nobody taught evolution in Tennessee back then! Somebody put him up to it, didn't they?

**Jones:** As I read it, Scopes, who was certainly pro-evolution himself, was kind of dragged into the fray and set up to teach evolution with the understanding that he would be defended. And the punishment that he was exposed to was essentially fines, so there wasn't much risk to Scopes, and of course, the benefit to Scopes was that he would be the centerpiece of this spectacular trial.

The marked difference, for historical purposes, is that Clarence Darrow, who represented Scopes, wanted to inject some scientific testimony into the trial, and the trial judge would not allow that testimony. So, it was really on the statute itself—did Scopes violate the statute itself?

**Gitschier:** Which he did.

**Jones:** He did. And the most memorable moment, as you may recall, is when Darrow called his opposing council, William Jennings Bryan, as a witness. That would never happen today. Bryan didn't have to take the stand, even then, but filled with excessive hubris, he took the stand and was eviscerated and embarrassed by Darrow. And the post-script was that Bryan died within a week of the trial.

**Gitschier:** I'm having trouble figuring out why we keep having this battle about fundamentalist beliefs in our public schools. I keep asking the question: Didn't we solve this problem already?

**Jones:** No.

**Gitschier:** And cutting to the chase, *have* we now solved the problem?

**Jones:** No.

**Gitschier:** OK, so let's talk through some of the background and figure out why not.

**Jones:** Scopes took place 80 years ago, and the matter was fairly dormant after that.

**Gitschier:** Why?

**Jones:** For decades afterwards, evolution was not substantially taught or taught at all.

**Gitschier:** In Tennessee or anywhere?

**Jones:** Anywhere. But by the '50s in the US, with Sputnik and the Cold War, there was a belief that we were falling drastically behind in science education and in other things, and you began to see a much more dedicated science component of education.

However, in certain pockets of the United States, particularly the South, there were anti-evolution statutes still on the books, and starting in the late 1960s, there was a progression of cases…

**Gitschier:** Starting with Epperson v. Arkansas?

**Jones:** Well, Susan Epperson's case. Susan was a young biology teacher who was involved in a lawsuit that had to do with a law prohibiting the teaching of evolution.

It was the same thing as Scopes, but now we're going to go after the *statute* itself. And Susan, whom I've met—a marvelous woman—was the prototypical plaintiff. She was a person of faith. She was young. She was telegenic, articulate, and she agreed to be the plaintiff in that case, which went all the way to the Supreme Court of the United States.

The result of Epperson was that a law that banned the teaching of evolution is struck down.

**Gitschier:** That law was in the State of Arkansas, and it was ruled that the Arkansas statute on the banning of teaching evolution was unconstitutional. Did that immediately, though, translate to other state laws?

**Jones:** Yes. It didn't “translate” to other state laws, but the Supreme Court, the highest court in the land, had spoken. Not per se; it ruled on the statute it had before it. But to the extent that other statutes were analogous to the Arkansas statute, the ruling meant that the wind had gone out of them. You couldn't enforce them.

**Gitschier:** So did it mean that evolution was now taught in Alabama or Tennessee, for example?

**Jones:** Not necessarily. It was still up to the school board whether they wanted to teach it or not.

But then, what states did was this: They said, “OK fine, we understand that we can't prohibit the teaching of evolution,” so they developed what has been called a “balanced treatment” statute, which said that if you are going to teach evolution, then you have to teach creationism next to it.

The states said, “We must live with the Supreme Court's decision in Epperson v. Arkansas, so now we're going to try to figure a way around it and get the best deal we can. We'll hold our nose, we don't like this, but if we're going to teach evolution, we're going to teach creationism at the same time, as an alternative to evolution.”

You've got a succession of cases, and I'm not trying to be encyclopedic, but again the Court said, “You're not listening. You can't teach creationism and call it what it is not.”

**Gitschier:** These were federal court rulings?

**Jones:** These were all federal court rulings because they deal with the Constitution. Then following that was, “How about this, we'll have you teach ‘creation science’.” And after drilling into that, the Court [in Edwards v. Aguillard] said, “No, a studied examination of creation science indicates that it is nothing more than creationism labeled in a different way.”

**Gitschier:** So, once creationism or creation science is struck down in one case, then what happens to all the other places that teach creation science?

**Jones:** Well, when the Supreme Court of the United States speaks, they can't do it. The bottom line is that as that line of cases concluded, you knew that you couldn't ban the teaching of evolution, you knew that you couldn't pass a “balanced treatment” statute, and you knew that you couldn't re-label creationism as creation science and have it pass constitutional muster.

Which then set the stage for intelligent design.

**Gitschier:** I read that you learned about this suit on the radio while driving home from work one day.

**Jones:** I was leaving this courthouse in Harrisburg, and I heard on the news from a local radio station that a very large lawsuit had been filed. There was a press conference at the state capitol rotunda, right across the way, by the plaintiffs' attorneys and that the suit was an establishment clause.

**Gitschier:** When you say “large,” you don't mean financially large.

**Jones:** Large, meaning impactful, notable, involving a big issue. And lawyers for the plaintiffs, the ACLU and a firm from Philadelphia, Pepper Hamilton, and the plaintiffs all appeared in the capitol rotunda. And they said that the suit had been filed in the Middle District of Pennsylvania, which is my district, and I've joked since then that I had two thoughts then. One, although I consider myself reasonably well-read, I could not remember hearing about ID before, so I really didn't know what it was. And two, I wondered who would get the case. And then forgot about it until I got into my Williamsport Chambers the following morning and looked at my new cases.

**Gitschier:** How many Middle District judges might have seen the case?

**Jones:** At the time there were five of us. I got it by the luck of the draw. It rotates in a sequence. I'd like to tell you it's because I'm so good, but it was just random.

**Gitschier:** Tell us about your education for this case. Although you hadn't heard of ID, you likely had heard of creationism or creation science. Had this been a field that you followed at all?

**Jones:** No, not other than popular culture. When I went to law school in the late '70s, I followed the progression of cases that we talked about before. I understood the general theme. I'd seen *Inherit the Wind*.

**Gitschier:** So now it's on your docket, and you must have been curious. Did you Google intelligent design?

**Jones:** No. I got what I needed in the context of the case. And it was the monster on my docket.

To your question: I think laypersons apprehend that when we get a case, it's incumbent upon us to go into an intensive study mode to learn everything about it. Actually that is the wrong thing to do. The analogy is that when I have a jury trial in front of me, I always instruct jurors, particularly in this day and age when you can Google anything, not to do that. I don't want you to do any research or investigation. Everything you need to decide this case you'll get within the corners of this courtroom.

So it is with me. And I knew that by the time the case went to trial and during the trial, that I would get expert reports.

**Gitschier:** From whom?

**Jones:** Everybody. The way expert opinion works is that I get a summary of their testimony first, and that I can read in advance. So I have a flavor for it. So then the question is, why also have them testify? That is because they are subject to cross examination and everything they say may not hold up that well. And, as it turned out, some of it didn't during the trial.

In any event, I was taken to school. From the earliest point in the litigation to the time the briefs were filed, it was the equivalent of a degree in this area. Folks who disagree with my opinion will tell you I never got it right, but I'm confident that I did.

Go back to your last question. It's very critical. I have to decide cases on the facts that are before me. I can't decide a case based on my own opinion, gleaned from outside the courtroom. That's why I don't engage in my own independent investigation. If you look at other systems in other countries throughout the world, they *do* that. But in our system of justice in the US, we let the parties try their cases and we find the facts from what is presented to us in the courtroom. And the law, presumably we know and we apply the law. That's our job. But the facts that we apply the law to are covered at that time.

**Gitschier:** I don't know if you're even allowed to answer this. Before this case landed on your lap, did you have any thoughts about creationism or evolution, or the debate?

**Jones:** The precursor to my answer is that it doesn't matter. A judge could be an avowed creationist, but he's got to rule based on the facts and the law. In that event, he'd have to hold his nose and do his duty as a judge.

I am a person of faith. I'm certainly not an atheist or an agnostic and I see some divine force somewhere. That said, having had a pretty good education, a great liberal arts education at Dickinson College, I must say that I never had any substantial doubts about evolution generally. I had forgotten, admittedly, a lot of what I had learned about evolution back in college. Moreover, a lot had happened since the '70s, so my understanding was rudimentary. But I never had a crisis of confidence about evolution or a reason to doubt that it constituted a valid theory and good science.

**Gitschier:** Regarding the Memorandum Opinion itself, I found parts of it astonishing. You used words like “mendacity,” “sham,” “breath-taking inanity of the board's decision.”

**Jones:** You should have been there.

**Gitschier:** I wish! Going into this you are impartial. What were some of the highlights? What were the transformational points in the trial that then allowed you to say, “OK, I'm going to rule this way”?

**Jones:** I don't think there was an epiphany. The very first witness for the plaintiffs was Ken Miller. He is very invested in this issue. He writes a textbook that is used substantially in high school biology classes throughout the country. And I think it's fair to say that the plaintiffs knew what they had in terms of their judge. They knew that I was not a scientist, but hopefully that I had a reasonably good head on my shoulders, that they were going to need to take me to school. So their first witness did just that.

I will always remember Ken Miller's testimony in the sense that he did A–Z evolution. And then got into intelligent design. And having laid the foundation with the description of evolution, got into why intelligent design doesn't work as science, to the point where it is predominantly a religious concept.

**Gitschier:** Is the other side objecting all the time?

**Jones:** They can object to a question that is truly objectionable. But there weren't a huge amount of objections. I let both parties try their case. They knew they'd have their turn.

Which gets me to the next point. Another remarkable moment on the science side was Michael Behe, who was the lead witness for the defendants, and a very amiable fellow, as was Ken Miller, but unlike Miller, in my view, Professor Behe did not distinguish himself. He did not hold up well on cross-examination.

So on the science side those were the two remarkable witnesses, although there were many expert witnesses in the field of theology, paleontology, biology, pedagogy.

**Gitschier:** It's almost like a command performance! There's no jury, it's not televised. All of these knowledgeable people…

**Jones:** Playing to an audience of one. Which was fascinating.

In the realm of the lay witnesses, if you will, some of the school board witnesses were dreadful witnesses and hence the description “breathtaking inanity” and “mendacity.” In my view, they clearly lied under oath. They made a very poor account of themselves. They could not explain why they did what they did. They really didn't even know what intelligent design was. It was quite clear to me that they viewed intelligent design as a method to get creationism into the public school classroom. They were unfortunate and troublesome witnesses. Simply remarkable, in that sense.

**Gitschier:** Did Miller talk about molecular evolution, DNA sequences, etc.?

**Jones:** To the extent that he needed to.

**Gitschier:** Because the evidence *is* amazing.

**Jones:** It is stunning when you get into it. Broadly, as the trial progressed, what was remarkable to me, as you go back—you well know this in your field—people called it Darwin's theory of evolution. Here's Charles Darwin, who had not the benefit at all of genetics, and yet from my view, almost every subsequent discovery tends to bear out Darwin's theory and has only made it stronger, including the field of genetics. But Ken Miller went into the immune system, the blood clotting cascade, and the bacterial flagellum—all three are held out by intelligent design proponents as irreducibly complex, and in effect, having no precursors. He [Miller] knocked that down, I thought, quite effectively—so comprehensively and so well. By the time Miller was done testifying, over the span of a couple of days, the defendants were really already in the hole.

But I can't decide the case until I hear all the evidence, and I didn't.

**Gitschier:** I want to address a very specific part of your Memorandum Opinion, which is defining science. What were you trying to do here?

**Jones:** First of all, both sides presented ample scientific testimony, and they asked me to decide that.

**Gitschier:** Both parties wanted you to address the question of what is science?

**Jones:** Well, not what is science, but whether intelligent design is science. Why else would they have presented all those expert witnesses?

**Gitschier:** Do they explicitly say that?

**Jones:** Sure they do.

**Gitschier:** Is that part of the original suit?

**Jones:** Yes, part of the analysis—the second prong of the Lemon test and the collapsed endorsement test [see Sidebar (Box 1)]—is the effect on the intended recipients. My view, and I'll always believe that I was right about this until somebody convinces me otherwise, is that if you're going to measure the effect of a particular policy, in this case juxtaposing intelligent design with evolution, on the intended recipients, you have to delve into what the policy is about. What was it about? It was about intelligent design. And to try to determine the effect on the recipients you have to determine what does that concept or phrase stand for? Hence, we got into a search and examination of what exactly does ID say, what is its basis, what are its scientific bona fides or lack thereof. That opens the door for a determination of whether ID is in fact science. And that is what that part of the opinion was.

People shouldn't mischaracterize it and say that I am the arbiter of what science is broadly. It's not what I wrote about in the opinion. I wrote about whether ID, as presented to me, in that courtroom from September to November of 2005, was science, and I said it was not. That it was the progeny, the successor to creationism and creation science. That it was dressed-up creationism.

**Gitschier:** Nonetheless, you have captured the essence of science in your opinion.

**Box 1. Sidebar: The Judge Provides a Primer**

**Gitschier:** There are a number of things in your Memorandum Opinion that I want you to flesh out for our readers. One is the Establishment Clause of the First Amendment. Second is the Lemon test and the prongs of the Lemon test. And the third thing that I really wasn't clear on was the endorsement test.

**Jones:** Lot of lawyers aren't clear on that either; it's very complex.

The Establishment Clause as contained in the First Amendment, simply stated, says that Congress shall pass no law that, in effect, favors an established religion. It's been the subject of a great deal of debate. Initially, in its inception, it was applicable only to Federal government, but with the Fourteenth Amendment, it was made applicable to the states, and hence, applicable to any governmental or quasi-governmental body including a school board. So there is no debate that the school board was subject to strictures of the establishment clause of the First Amendment.

There is a vigorous debate that takes place, to this day, as to whether there is a wall of separation between church and state, as Thomas Jefferson opined. That phrase doesn't appear anywhere in the Constitution. However, the Supreme Court of the United States has clearly set out, in its decisions over the past 60 years, that there is a wall, porous at times, but a wall nonetheless. So the common theme of their decisions is that they are going to look with a high degree of scrutiny on government activity that seems to favor a particular religious concept. Hence the line of cases we talked about before.

Now the devil is in the details, and so it then fell to the Justices to develop, as they typically do in cases like this, tests—overlays, if you will—that they put against the facts that are found by judges, so that the judges can decide whether a violation has occurred. As you might expect, because every case is so intensively fact-specific, sometimes these tests are really hard to apply.

So the first test that the Court came up with is the Lemon test, Lemon v. Kurtzman [another Pennsylvania case regarding the reimbursement of Catholic schools by the state superintendent of schools].

What came out of Lemon were three prongs that judges have to look at. The first is: what is the purpose of the enactment? The second is: what is the effect of the enactment? And the third is: is there an excessive entanglement between religion and government?

I'll come back and be specific to my case [in a minute]. As time went by, it was apparent that the Lemon test was somewhat difficult to apply in certain factual situations. In particular it was found to be difficult to apply in cases where, for example, the Ten Commandments were bolted onto the side of a courthouse or government building. So Former Justice Sandra Day O'Conner then penned the “endorsement test.” The endorsement test, boiled down to its essence, takes the first two prongs—the purpose and the effect prongs—and collapses them together, and just makes it easier to apply, although it is always hard to judge these cases.

To go back to the Lemon test. If the judge finds that the purpose is predominantly religious, you can stop; you don't have to go to the other prongs. But if you find it's OK, you can go to the effect prong—what is the effect on the intended recipients of the policy? How do they view it? If you find a violation there, you needn't go to the excessive entanglement prong.

In my case [Kitzmiller], it failed the purpose prong, and the excessive entanglement prong was never at issue, by agreement of counsel on both sides. But for the sake of completeness, because I had to believe that my decision would be appealed, I did the effect prong as well. And I also did the endorsement test. But the endorsement test is just a variation on the Lemon test, and is in some ways a duplication of the Lemon test, with a twist.

**Jones:** Well, you could read it that way if you chose to. What it does contain is something that you could utilize as a portable mechanism to look at other concepts and decide whether they were science. But the question I decided was whether ID was science. And you use tools like—is it testable? Is it peer reviewed? Is it generally accepted in the scientific community? And the answer to all three of those things is “No.”

**Gitschier:** Let's talk about what happened downstream of this decision. How will this change affect the landscape of education in the US?

**Jones:** The short answer is that I don't know. In the two and a half years since the opinion was released, no one has tried to teach ID in the US. Remember, the opinion doesn't have precedential effect outside of Pennsylvania. In other words, I am a Federal District court with jurisdiction over this big middle of Pennsylvania, but I'm not the Supreme Court of the United States. So, it's unlike the mandates from the Supreme Court that we were discussing earlier such as Epperson and Edwards. Those are the final words for now, and everyone must adhere to them. I suppose a school board in another state could still pass a law mandating the teaching of ID, and in fact some were considering doing so at the time of this trial, but later pulled them down. But I do think that many consider my opinion persuasive, if not binding, and that's why you have not seen these policies enacted.

**Gitschier:** Such as in Kansas?

**Jones:** Such as in Kansas. Kansas at that time was having [state-wide] school board elections. And this became an issue in Kansas, and Kansans did not elect proponents of ID, utilizing my decision I think, saying that it was improvident to do this. In Ohio, they had begun steps that would have allowed the teaching of ID, and the school board ruled the policy back because of my decision, not because they had to, but they thought it was persuasive. Florida had a debate last year, into this year about changing some of their standards or adopting new standards of science, again citing my decision.

The hotbeds today—and this is re-emerging—Texas has a very strong desire to get into something like teaching intelligent design. Louisiana just passed a stature that seems like it could be used as a vehicle for teaching ID. This is speculation on my part—I don't think that the concept of ID itself has a lot of vitality going forward. The Dover trial discredited that thing that is ID. To the extent that I follow it—I'm curious about it, but it doesn't go any further than that—the likely tack going forward is something like teach the *controversy*, talk about the alleged flaws and gaps in the theory of evolution and go to that place first.

They gave me the last word in “Judgment Day” [a NOVA program on the trial] and I said this is not something that will be settled in my time or even in my grandchildren's lifetimes. It's an enduring, quintessentially American, dispute. If you poll in the US today, you'll find that approximately half of our fellow citizens believe in creationism and think that creationism ought to taught.

**Gitschier:** I had no idea!

**Jones:** Believe me. Remember, the Dover School Board was comprised of young-earth creationists. They believe that the Bible is the Word. They either can't explain or like not to explain the evidence to the contrary. Then there are the mixed-bag creationists—creationists who accept that the world is as old as it is but don't accept evolutionary mechanism.

**Gitschier:** How has this trial changed your life? Both externally and in the way you think about the world.

**Jones:** It's changed my life forever. You can't go through something like this that has such notoriety without being changed. Federal Judges at any level lead quite cloistered existences, and I was thrust onto the stage in a way that I would never have thought possible. And I have been speaking all around the US, but I don't go and try to say what I did in the opinion.

What I developed was a passion for the concept known as “judicial independence,” meaning that concomitantly with the science education issue that I just raised, I don't think Americans understand how judges operate.

I had a lot of criticism after this decision; a lot, I think, was born out of ignorance about how we do things. People didn't understand there was a Lemon test or an Endorsement test. People thought I made this up as I went along. They think judges rule according to their own philosophical biases or predilections. I thought it was incumbent upon me to get out and talk about that and say, “Well, you don't quite have this right,” and I've been very well received across the country.

But from the NOVA show to the now four books that have been written about the case, to being on the cover of Time magazine, for someone born and raised in a town of 2,000 in upstate PA—all this is fairly miraculous stuff that I never thought I would do. So, it certainly has changed the fabric of my life, that I have had this interval. It will die down, I know.

When I take my last breath and they publish my obituary, the first line will say that I presided over the intelligent design trial. I can't *top* this, I don't think, and I'm fine with that, if this is what I'm remembered for. I'm proud of what I did. I thought I discharged my obligations and my duties well.

Going forward, has it made me more curious about the issue? Yes, and I think I'll always have that enduring curiosity.

Recommended reading from the Judge:

- *Summer for the Gods* by Edward J. Larson;
- *The Devil in Dover* by Lauri Lebo;
- *40 Days and 40 Nights* by Matthew Chapman.

